# Early Auditory Event-Related Potentials Are Modulated by Alphabetic Literacy Skills in Logographic Chinese Readers

**DOI:** 10.3389/fpsyg.2021.663166

**Published:** 2021-07-29

**Authors:** Yubin Zhang, Chotiga Pattamadilok, Dustin Kai-Yan Lau, Mehdi Bakhtiar, Long-Ying Yim, Ka-Yui Leung, Caicai Zhang

**Affiliations:** ^1^Department of Linguistics, University of Southern California, Los Angeles, CA, United States; ^2^Laboratoire Parole et Langage (LPL), CNRS, Aix Marseille University, Aix-en-Provence, France; ^3^Department of Chinese and Bilingual Studies, The Hong Kong Polytechnic University, Hong Kong, China; ^4^Unit of Human Communication, Development, and Information Sciences, The University of Hong Kong, Hong Kong, China; ^5^Research Centre for Language, Cognition, and Neuroscience, The Hong Kong Polytechnic University, Hong Kong, China

**Keywords:** phonological awareness, alphabetic orthography, auditory onset phoneme judgment, event-related potentials, Chinese

## Abstract

The acquisition of an alphabetic orthography transforms speech processing in the human brain. Behavioral evidence shows that phonological awareness as assessed by meta-phonological tasks like phoneme judgment, is enhanced by alphabetic literacy acquisition. The current study investigates the time-course of the neuro-cognitive operations underlying this enhancement as revealed by event-related potentials (ERPs). Chinese readers with and without proficiency in *Jyutping*, a Romanization system of Cantonese, were recruited for an auditory onset phoneme judgment task; their behavioral responses and the elicited ERPs were examined. Proficient readers of *Jyutping* achieved higher response accuracy and exhibited more negative-going ERPs in three early ERP time-windows corresponding to the P1, N1, and P2 components. The phonological mismatch negativity component exhibited sensitivity to both onset and rhyme mismatch in the speech stimuli, but it was not modulated by alphabetic literacy skills. The sustained negativity in the P1-N1-P2 time-windows is interpreted as reflecting enhanced phonetic/phonological processing or attentional/awareness modulation associated with alphabetic literacy and phonological awareness skills.

## Introduction

The functional organization of the human brain can accommodate itself to new experiences. As a recent cultural invention in human history, the writing system of language influences how the brain represents and processes phonological information in a significant way (Castro-Caldas et al., 1998; Perre et al., 2009; Dehaene et al., 2010, 2015; Monzalvo and Dehaene-Lambertz, 2013; Morais, 2021).

One example of such influence is that the grain size of phonological representations and processing has been suggested to be shaped by the units employed in the orthographic system (Ziegler and Goswami, 2005). In an alphabetic writing system such as English, Dutch or French, the orthographic letters represent speech sounds at a more fine-grained phonemic level. However, in a logographic system like Chinese, the characters are associated with phonology at a much coarser grain size, i.e., the morpho-syllabic level (Perfetti et al., 2010). Although a significant portion of compound Chinese characters contains phonetic radicals that can be employed for retrieving phonological information, the phonetic radicals refer to the pronunciation of the whole syllable instead of individual phonemes (Perfetti and Tan, 1998). Phonological awareness and representations at the relatively fine-grained abstract phonemic level have been argued to be heavily influenced by the acquisition of an alphabetic writing system. Some researchers even argue that the phoneme is a literate cognitive concept induced by alphabetic literacy acquisition (Morais et al., 1979; Read et al., 1986; Morais, 2021). Now, prior to the introduction of the current study, we turn to a brief overview of previous neural and behavioral evidence on the influences of learning an alphabetic writing system on spoken language representations and processing.

### The Impact of Acquiring an Alphabetic Script on Speech Representations and Processing: Behavioral and Neural Evidence

The most prevalent behavioral paradigms to investigate speakers’ representations and awareness to phonological units are tasks that tap meta-phonological skills, such as phoneme deletion/addition (Morais et al., 1979; Read et al., 1986; de Gelder et al., 1993), pseudoword repetition (Reis and Castro-Caldas, 1997), auditory onset/rhyme judgment (de Gelder et al., 1993), sound matching or detection (Ho and Bryant, 1997; Cheung et al., 2001), and phoneme counting (Liberman et al., 1974). Performance on the identification and manipulation of phonological units in such tasks is considered as an index of phonological awareness to speech units with different grain sizes. Speakers’ phonemic awareness is evaluated when the task focuses on phonemic manipulation.

Earlier behavioral evidence showed that compared with proficient readers of an alphabetic script, preliterate children have difficulties with explicit phoneme manipulation, such as counting the number of phonemes in speech stimuli (Liberman et al., 1974). However, this difference in performance could also be due to differences in the maturation of general cognitive skills between the two populations.

Later work investigated phonological awareness in literate and illiterate adults in an alphabetic script (Morais et al., 1979; Reis and Castro-Caldas, 1997). Similar difficulties with explicit phonemic analysis have been observed in illiterate adults even though their speech and general cognitive systems have reached maturity. Illiterates were found to show lower performance than their literate peers in tasks tapping explicit phonemic analysis, such as phoneme addition/deletion (Morais et al., 1979) and pseudoword repetition (Reis and Castro-Caldas, 1997).

Another line of behavioral evidence comes from studies that focus on phonological awareness in readers with different levels of knowledge of logographic versus alphabetic scripts (Read et al., 1986; de Gelder et al., 1993; Ho and Bryant, 1997; Cheung et al., 2001; Shu et al., 2008). In an early study, Read et al. (1986) reported that Chinese readers who were literate only in the logographic script exhibited poorer phonemic awareness than readers with additional knowledge of *Pinyin*, which is an alphabetic script of Mandarin Chinese. More recently, Shu et al. (2008) investigated the development of phonological awareness in young Chinese children in a series of sound detection tasks. Their results showed that the kindergarten children could not perform the onset phoneme detection task above chance level whereas the first graders with *Pinyin* training achieved 70% accuracy, suggesting that some levels of phonological awareness especially onset phonemic awareness is dependent on alphabetic literacy. However, since this study did not include first graders without *Pinyin* instruction, we cannot completely exclude the possibility that the better performance in onset detection for the first graders was due to their better general cognitive skills and more experience with the ambient spoken language. In a cross-linguistic developmental study, Ho and Bryant (1997) found that Cantonese-speaking Chinese children in Hong Kong developed consonant awareness later than their English counterparts in primary school. Cheung et al. (2001) compared the phonological awareness skills of Hong Kong Cantonese-speaking children who had knowledge of the logographic Chinese script only and Guangzhou Cantonese-speaking children who received training in both logographic Chinese and *Pinyin* scripts. They found that Guangzhou children performed better than their Hong Kong counterparts in onset and coda matching, suggesting the influences of alphabetic knowledge on phonemic awareness.

Further behavioral evidence comes from other behavioral paradigms, like word-form encoding in production and primed-naming experiments in the visual modality (Perfetti and Bell, 1991; Perfetti and Tan, 1998; Chen et al., 2002). These experiments do not require explicit phonemic manipulation but still provide insight on the cross-linguistic differences in the grain size of phonological representations associated with orthographic systems. In an implicit priming study on word-form encoding in Chinese, Chen et al. (2002) found that both syllable+tone and syllable-alone units evoked implicit priming effects, whereas the syllable onset unit (corresponding to a phoneme) did not. In contrast, onset priming was detected in Dutch speakers using a similar paradigm (Meyer, 1991). The discrepancy suggests phonemic level encoding in Dutch but not in Chinese (Chen et al., 2002). In Chinese character primed-naming experiments, Perfetti and Tan (1998) reported that facilitatory homophonic (phonological) priming effects, e.g., for a prime-target pair like “其 (*Pinyin*: qí) – 齐 (qí),” occurred relatively late, when the early facilitatory graphic priming effects, e.g., “何 (hé) – 向 (xiàng),” became inhibitory. The authors suggested that in Chinese readers, graphic and phonological information are activated independently in distinct cycles, and phonological activation arises once orthographic processing has been completed, presumably without phonemic assembly. These results are in contrast to those obtained from an earlier priming study in English readers (Perfetti and Bell, 1991). In that study, the target word (e.g., *rate*), was presented visually after three types of pseudoword primes–phonemic prime (e.g., *rait*), graphemic prime (e.g., *ralt*), and control prime (e.g., *busk*). The results revealed early and synchronously increasing facilitatory effects of both homophonic and graphic primes, thus suggesting that phonological activation arises synchronously with the processing of the constituent graphic units.

Neuroimaging studies using tasks that require meta-phonological skills have revealed multiple neuro-cognitive networks that are sensitive to alphabetic and phonological awareness skills (Castro-Caldas et al., 1998; Burton et al., 2000; Petersson et al., 2000; Brennan et al., 2013). The left-dominant peri-sylvian language network, including the inferior frontal gyrus (IFG), superior temporal gyrus (STG), and planum temporale (PT) that are suggested to be responsible for phonetic/phonological processing, has been frequently found to be implicated in tasks that require meta-phonological analysis and literacy-induced changes in speech and language processing (Castro-Caldas et al., 1998; Burton et al., 2000; Petersson et al., 2000; Dehaene et al., 2010, 2015; Brennan et al., 2013; Monzalvo and Dehaene-Lambertz, 2013). Moreover, the attentional network involving the anterior cingulate cortex (ACC) and the central executive network including the prefrontal cortex, have been shown to be engaged in such tasks, and to vary in activation levels according to alphabetic literacy and phonological awareness skills (Castro-Caldas et al., 1998; Burton et al., 2000; Petersson et al., 2000).

Burton et al. (2000) conducted an fMRI study to examine the contribution of the frontal cortices during auditory phoneme judgment under different phonemic segmentation requirements. Participants were asked to perform “same/different” judgment on the onset consonants of English word pairs presented in two lists— “global” and “segmental”. In the global list, where the two words in a pair always shared the same rhyme in a word pair (e.g., *dip-tip* and *dip-dip*), participants were expected to adopt a “global strategy” that consists in making judgments without segmenting out the onset phonemes. Conversely, in the segmental list where the rhymes differed (e.g., *dip-tomb* and *dip-doom*), separating the onset phonemes from the rest of the words was assumed to be mandatory. The imaging results indicated that the STG, subserving phonetic/phonological processing, was activated for both lists. However, only the segmental list elicited activations in frontal cortices (left IFG and middle frontal gyrus), which the authors interpreted as increased demands of phonemic segmentation, articulatory recoding or working memory in the segmental list. Stronger ACC activations were also found in the segmental list. This result possibly reflected increased attentional modulation or conflict monitoring because more attentional resources might be needed in the segmental list to focus on the target onset dimension and to ignore or inhibit the irrelevant rhyme differences in a word pair.

A recent fMRI study by Brennan et al. (2013) investigated the developmental differences in the phonological network in Chinese and English speakers using an auditory rhyme judgment task. The imaging results revealed developmental increase in the activation of the left STG, IFG, and inferior parietal lobule (IPL) for English speakers only. This is taken as evidence that literacy acquisition leads to the reorganization of the phonological network for speakers of a language with an alphabetic writing system only.

### The Current Study

While there is a large body of literature examining the neuro-cognitive networks in meta-phonological tasks, the temporal dynamics of these cognitive operations underlying phonological awareness skills during phoneme judgment is still unclear. More importantly, it remains to be studied how the time-course of these neuro-cognitive events in phonemic processing can be influenced by the acquisition of an alphabetic script. Existing ERP studies using meta-phonological tasks were not designed specifically for this question (McPherson et al., 1998; Pattamadilok et al., 2011; Lafontaine et al., 2012). For example, Lafontaine et al. (2012) employed an auditory onset judgment task and manipulated the congruency between the phonological and orthographic information of the onsets in a French word pair. Their goal was to investigate the time-course of phonological and orthographic activations. As such, there remain research gaps regarding the ERP indices of how learning an alphabetic system affects the time-course of the neuro-cognitive operations underlying phonological awareness skills required in meta-phonological tasks.

The major goal of the present ERP study is to identify the speech processing stages and the corresponding neuro-cognitive operations involved in phonemic processing that are sensitive to alphabetic literacy skills in adult readers of logographic Chinese. To this end, we recruited two groups of Cantonese speakers with and without proficiency in *Jyutping*, a romanization system of spoken Cantonese, for an auditory onset judgment task. One motivation for choosing *Jyutping* is that it allows us to investigate whether and to what extent the relatively late acquisition of an alphabetic script influences the neuro-cognitive processes underpinning phonological awareness skills in adult readers of logographic Chinese. In Mainland China, *Pinyin* is taught to children at the same time as they begin to learn the Chinese logographic writing system. However, Cantonese children in Hong Kong learn the logographic Chinese writing system without the assistance of a native alphabetic script. Some Cantonese speakers choose to learn *Jyutping* primarily for the purpose of inputting Chinese on social media. Although most Cantonese speakers in Hong Kong have some acquaintance with the alphabetic English script, it remains controversial whether learning to read in an alphabetic script of a second language like English would influence their phonological processing in Cantonese in the same way and to the same extent as acquiring an alphabetic script of their native language Cantonese (Bialystok et al., 2005; Dodd et al., 2008; Deng et al., 2019).

Besides the between-subject manipulation of *Jyutping* knowledge (i.e., the *group* factor), the speech stimuli employed in the auditory onset phoneme judgment task were divided into two lists–global and segmental–as in Burton et al. (2000) to manipulate the segmentation demands. The paired Cantonese pseudoword stimuli in the global list always contained the same rhyme (e.g., paa^2^-paa^2^ and paa^2^-faa^2^, transcribed in *Jyutping*) whereas the stimuli in the segmental list contained different rhymes (e.g., paa^2^-paai^2^ and paa^2^-faai^2^). This *list* manipulation allowed us to test whether the alphabetic knowledge affects speech processing situations with different “segmentation” demands in the same way (Burton et al., 2000). Finally, for the purpose of the same/different onset phoneme judgment task, within each list, pairs of stimuli with both congruent and incongruent onsets were included in the materials. Altogether, these manipulations led to a 2 × 2 × 2 design with three factors: *group* (non-*Jyutping* vs. *Jyutping*), *list* (global vs. segmental) and *onset congruency* (congruent vs. incongruent).

For behavioral responses, we predicted that the group with *Jyutping* proficiency would achieve higher accuracy and respond more quickly than the group with low *Jyutping* proficiency (Read et al., 1986; de Gelder et al., 1993; Ho and Bryant, 1997; Cheung et al., 2001; Shu et al., 2008). Furthermore, the segmental list would elicit less accurate and slower responses than the global list (Burton et al., 2000). The group effect could also be amplified by task difficulties caused by the characteristics of the list or onset congruency.

Regarding the predictions on ERP responses, a growing number of studies have shown that more efficient auditory or speech/musical processing abilities are associated with an enhancement of early auditory evoked potentials like N1-P2 (Tremblay et al., 2001; Tremblay and Kraus, 2002; Baumann et al., 2008; Shahin, 2011). Since acquiring an alphabetic script leads to the improvement of speech processing abilities (Read et al., 1986; Castro-Caldas et al., 1998; Brennan et al., 2013; Dehaene et al., 2015), we could reasonably expect the participants with *Jyutping* expertise to show higher amplitude in the N1 and P2 components than those without *Jyutping* proficiency. Additionally, the impact of the knowledge of a native alphabetic script might also be found at a later phonological processing stage as indexed by the phonological mismatch negativity (PMN). A prevalent view is that the PMN reflects the stage of translating acoustic properties of speech sounds into phonemic candidates, and this process is affected by the mismatch between phonemic expectations in the working memory established by prior contexts and the actually presented speech signals (Connolly and Phillips, 1994; Newman et al., 2003; Lafontaine et al., 2012; Zhang, 2018). In the auditory phoneme judgment task, the PMN component would reflect an active detection of a mismatch in phonological units, like onsets and rhymes, in a pair of speech stimuli (Lafontaine et al., 2012). For example, in our design, two types of phonological mismatch would be expected–one induced by the manipulation of *onset congruency*, which was the target dimension of the task, and the other induced by the *list* manipulation, which might engender additional phonological mismatch at the rhyme level. If the PMN is modulated by alphabetic literacy skills, the ERP amplitude in this time-window would be affected by the interaction between *group* and *list* or between *group* and *onset congruency.*

## Methods

### Participants

Twenty-four logographic Chinese readers with *Jyutping* competency (henceforth, the *Jyutping* group) and twenty-two participants without *Jyutping* competency (henceforth, the non-*Jyutping* group) participated in the study. The two groups of participants were determined based on their *Jyutping* proficiency, which was evaluated by a timed *Jyutping* transcription test. The *Jyutping* test included 20 disyllabic Chinese words presented to the participants as 40 Chinese characters, which covered as many onsets and rhymes in Cantonese as possible. Participants were required to transcribe the 40 Chinese characters into *Jyutping* labels within 2 minutes (e.g., from “變化” to bin^3^ faa^3^). In line with the aim of the study, participants’ scores for transcribing onset consonants were calculated (40 points in total). The *Jyutping* group obtained much higher average scores for onset phoneme transcription than the non-*Jyutping* group (mean ± SD: 29.7 ± 8.3 vs. 7.5 ± 3.7). In both groups, the majority of the participants had completed or had been pursuing a college degree at the time of participation and none of them majored in linguistics or psychology. The two groups were matched for age, English proficiency (standardized English scores), and musical experience. Their English proficiency was evaluated based on their most recent scores obtained in standardized English tests used in Hong Kong (IELTS, HKDSE-ENG, or HKALE-ENG). Most of the participants were right-handed and all of them had no reported hearing, speech, or language disorders. The detailed demographic information of the two groups is presented in Table 1.

**TABLE 1:** Participant information.

	*Jyutping*	Non-*Jyutping*
Total	24	22
Gender (number of male/female)	17/7	13/9
Age (mean ± SD)	22.4 (±4.2)	21.9 (±2.8)
*Jyutping* scores (mean ± SD; total: 40)	29.7 (±8.3)	7.5 (±3.7)
Standardized English scores^1^ (mean ± SD)	6.8 (±0.6)	6.5 (±0.6)
Musical training^2^ (No. of participants)	3	3
Education level (No. of participants with a bachelor’s degree or higher)	22	19
Handedness (No. of right-handed participants)	23	22

*^1^A participant’s standardized English score was calculated from the International English Language Testing System (IELTS), Hong Kong Diploma of Secondary Education Examination-English Language (HKDSE-ENG), or Hong Kong Advanced Level Examination-English Language (HKALE-ENG), respectively. To make the standardized English tests scores comparable across participants, the HKDSE-ENG and HKALE-ENG scores were converted into equivalent IELTS band scores based on the results of a benchmarking study comparing IELTS and HKDSE-ENG/HLALE-ENG by the Hong Kong Examinations and Assessment Authority (HKEAA).*

*^2^Participants who have received musical training in either musical instrument or vocal singing for 3 years or longer were considered to be participants with musical training.*

Informed written consent was obtained from the participants in compliance with the experiment protocols approved by the Human Subjects Ethics Sub-committee of the Hong Kong Polytechnic University and with the 1964 Helsinki declaration and its later amendments or comparable ethical standards.

### Materials

The auditory phoneme judgment task included 96 Cantonese pseudoword pairs in spoken forms, which were constructed from six sets of Cantonese pseudowords (see Table 2). Each set contained four monosyllabic pseudowords, which are phonotactically plausible syllables, but their combinations with certain tones do not yield real words. For example, for the pseudoword “paai^2^,” “paai” is a legal Cantonese syllable, but combining this syllable with Tone 2 (high-rising tone) generates a pseudoword. Only Tone 1 (high-level tone), Tone 2 (high-rising tone), and Tone 4 (low-falling tone) were included as they are the most easily recognizable tones in Cantonese (Peng et al., 2012). Within each set, four syllables (e.g., paa^2^, paai^2^, faa^2^, and faai^2^) were formed by combining two onsets (e.g., p and f) and two rhymes (e.g., aa and aai), with the tone (e.g., tone 2) kept identical. For each of the six sets, 16 pseudoword pairs were constructed by combining each pseudoword with itself and then with the other three pseudowords (e.g., combining paa^2^ with other pseudowords–paa^2^-paa^2^, paa^2^-paai^2^, paa^2^-faa^2^, and paa^2^-faai^2^), resulting in a total of 96 pairs of pseudowords for all the six sets. The materials differed in two aspects–*list* (or rhyme difference, global vs. segmental) and *onset congruency* (onset difference, congruent vs. incongruent). Following Burton et al. (2000), the resulting 96 pseudoword pairs were divided into two lists, representing two experimental conditions–“global” and “segmental”. In the global list (48 pairs), two pseudowords in a pair had identical rhymes, whereas the onsets could be either congruent (e.g., paa^2^-paa^2^, paai^2^-paai^2^, faa^2^-faa^2^, and faai^2^-faai^2^) or incongruent (paa^2^-faa^2^, faa^2^-paa^2^, paai^2^-faai^2^, and faai^2^-paai^2^). In the segmental list (48 pairs), two pseudowords had different rhymes, whereas again, the onsets could be either congruent (e.g., paa^2^-paai^2^, paai^2^-paa^2^, faa^2^-faai^2^, and faai^2^-faa^2^) or incongruent (paa^2^-faai^2^, faai^2^-paa^2^, faa^2^-paai^2^, and paai^2^-faa^2^). The speech materials were recorded in a sound-attenuated booth by a female Cantonese speaker. The duration and mean intensity of the stimuli were normalized to 560 ms and 70 dB, respectively. Within each pseudoword pair, the inter-stimulus interval (ISI) was 440 ms.

**TABLE 2:** Six sets of auditory Cantonese pseudowords transcribed in *Jyutping* and IPA (in brackets).

Set 1	aa (/a:/)	aai (/a:i/)	Set 2	ei (/ei/)	eoi (/ey/)	Set 3	aau (/a:u/)	i(/iː/)
p (/p^*h*^/)	paa2	paai2	d (/t/)	dei4	deoi4	m (/m/)	maau2	mi2
f (/f/)	faa2	faai2	g (/k/)	gei4	geoi4	l (/l/)	laau2	li2

**Set 4**	**it (/it/)**	**aat (/a:t/)**	**Set 5**	**in (/i:n/)**	**eng (/εːŋ**	**Set 6**	**ek (/εːk/)**	**ok (/ɔːk/)**

c (/ts^*h*^/)	cit1	caat1	g (/k/)	gin4	geng4	t (/t^*h*^/)	tek1	tok1
l (/l/)	lit1	laat1	h (/h/)	hin4	heng4	s (/s/)	sek1	sok1

### Procedure

Participants were seated in an electrically-shielded booth. EEG signals were continuously recorded and digitized at a sampling rate of 2,048 Hz using a 32-channel ActiveTwo EEG system (BioSemi B. V., Amsterdam, Netherlands). The whole experiment was divided into two blocks, representing the global and segmental lists. The auditory speech stimuli were repeated twice, generating 96 trials for each list. A fixation cross, indicating the beginning of a trial, was presented at the center of the screen, and remained until the end of a trial. A pseudoword pair was presented binaurally through inserted earphones 2000 ms after fixation cross onset. Within each block, the auditory stimuli were presented pseudo-randomly. The participants were required to judge whether the two auditory speech stimuli in a pair shared the same onset by pressing the left or right button of the response box with their left or right index finger without a time limit. A manual response terminated a trial and the procedure proceeded to the next trial. The inter-trial interval was jittered between 1600 and 1800 ms.

### Data Analysis

The response accuracy and reaction time (measured from the onset of the second stimulus in a pair) were analyzed by a generalized linear mixed-effects model and a general linear mixed-effects model, respectively, using the *lme4* package in *R* (Bates et al., 2020). The raw reaction time (RT) was log-transformed to achieve closer approximation of normality of residuals. Both models included three deviation-coded (–0.5, 0.5) categorical variables–*group* (non-*Jyutping* vs. *Jyutping*), *list* (global vs. segmental), *onset congruency* (congruent vs. incongruent)–and their interactions as fixed effects. For the random-effects structures of both models, we began by including the by-participant random intercept only. Then, we tested whether including the other by-participant random slopes significantly improved model fit by the log-likelihood ratio test. According to the test results, the random slopes of *list*, *onset congruency* and their interaction were justified for both models. When an interaction effect was found, we also conducted post-hoc analyses using the *emmeans* package (Lenth et al., 2009).

The raw EEG data were preprocessed using EEGLab (version 2019). The raw EEG data were first band-pass filtered (0.1–30 Hz) and then segmented into 600 ms epochs, time-locked to the onset of the second pseudoword of a stimulus pair. The baseline was 100 ms before the onset of the second stimulus in a pseudoword pair and the post-onset interval was 500 ms. Baseline-correction was applied according to the 100 ms pre-stimulus interval. The epoched data were recalculated against the average-mastoid reference. Prior to Independent Component Analysis (ICA), we pruned bad epochs with non-stereotyped or paroxysmal artifacts which would affect the quality of ICA decomposition (Onton et al., 2006). This step led to a loss of 4.56% of the trials. After pruning, ICA was performed on the remaining data using the extended informax algorithm (Delorme and Makeig, 2004). Artifactual components, reflecting eye blinks and eye movements, were manually removed by inspecting the ICA topographies, waveforms and spectra. To calculate ERPs, the resulting epochs were averaged for each participant and each condition.

For the ERP analysis, we first calculated the global field power (GFP, i.e., spatial root mean square of voltage over all the electrodes) on the grand-averaged waveforms averaged across all the participants and conditions according to the following formula:

$${\text{GFP}}_{t}=\,\sqrt{\frac{\sum_{i\,=1}^{k}V_{i\,t}^{2}}{k}}$$

Where *t* is time, *k* is the total number of channels (*k* = 32), *V*_*it*_ is the voltage at channel *i* and time *t*.

**FIGURE 1:**
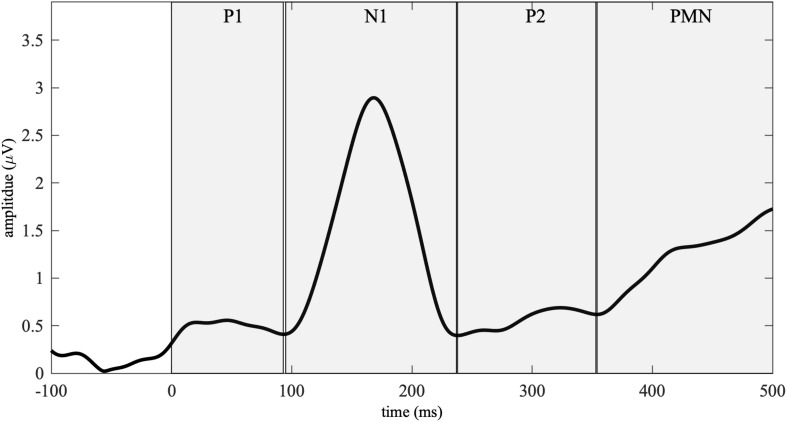
Global field power (GFP) calculated from the grand-averaged waveforms across all the participants and conditions. Four event-related potential (ERP) time-windows– (0–93 ms), N1 (95–237 ms), P2 (239–353 ms), and phonological mismatch negativity (PMN, 355–500 ms) were identified based on the GFP. The boundaries across time-windows (94, 238, and 354 ms) were defined as the minimal values from 50–150, 200–300, and 300–400 ms.

Based on the GFP and previous ERP literature (Connolly and Phillips, 1994; Tremblay and Kraus, 2002; Martin et al., 2008; Zhang, 2018), we analyzed the ERPs in four time-windows–P1 (0–93 ms), N1 (95–237 ms), P2 (239–353 ms), and PMN (355–500 ms, see Figure 1). ERP amplitude in the P1-N1-P2 time-window was analyzed by averaging the ERP waves from a fronto-central electrode cluster (F3, Fz, F1, FC1, FC2, and Cz; Näätänen and Picton, 1987; Tremblay and Kraus, 2002; Martin et al., 2008). The PMN analysis was based on the averages of ERPs from a centro-parietal electrode cluster (P3, Pz, P4, CP1, CP2, and Cz; Lafontaine et al., 2012). Since we did not predict hemispheric laterality effects, the electrodes were not further divided into left, midline, and right sites. Mean ERP amplitude of each time-window was calculated for each participant, list, and onset congruency condition, and was submitted to a three-way ANOVA, with three factors *group*, *list* and *onset congruency*. The ANOVA was conducted using the *afex* package in *R* (Singmann et al., 2020). When an interaction effect was detected, post-hoc analyses were conducted using the *emmeans* package (Lenth et al., 2009).

## Results

### Behavioral Results

**FIGURE 2:**
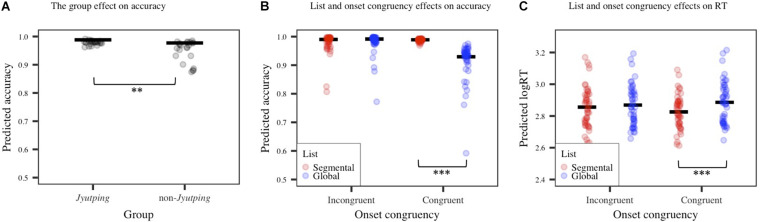
Accuracy and reaction time (RT) results. **(A)** The group effect on accuracy. **(B)** List and onset congruency effects on accuracy. **(C)** List and onset congruency effects on RT. The solid line denotes estimated means based on fixed effects while each dot represents individual fitted values. Significance levels: *p < 0.05; **p < 0.01; ***p < 0.001.

The behavioral results are displayed in Figure 2. The accuracy model revealed a significant main effect of *group* (β = 0.67, *z* = 2.47, *p* < 0.01). As expected, the *Jyutping* group achieved higher accuracy than the non-*Jyutping* group. Additionally, there was a significant main effect of *list* (β = –0.88, *z* = –5.13, *p* < 0.001), with more accurate responses elicited in the global list than in the segmental list. A significant main effect of *onset congruency* was also found (β = 1.14, *z* = 5.49, *p* < 0.001). There were generally more accurate responses for trials with incongruent onsets than congruent onsets, but the effect of *onset congruency* was further modulated by *list*, as shown by a significant interaction between *list* and *onset congruency* (β = 2.07, *z* = 5.72, *p* < 0.001). Post-hoc analysis revealed more accurate responses for the global list than the segmental list in trials with congruent onsets only (*z* = –10.88, *p* < 0.001). The results showed that the *congruent onset/segmental list* condition elicited the least accurate responses. The interactions between *group* and other factors were not significant (*p*s > 0.05).

The RT model revealed a significant main effect of *list* (β = 0.03, *t* = 4.23, *p* < 0.001), reflecting generally faster responses to the global list than to the segmental list, which is consistent with the accuracy results and confirmed our prediction that performing phoneme judgment for the segmental list is more challenging. This effect was modulated by *onset congruency* (β = –0.04, *t* = –6.4, *p* < 0.001). Post-hoc analysis revealed significantly faster responses for the global list than the segmental list in trials with congruent onsets only (*z* = 6.31, *p* < 0.001), which is similar to the pattern reported above on accuracy. No significant *group* effect or its interactions with other factors were found (*p*s > 0.05).

### ERP Results

**FIGURE 3:**
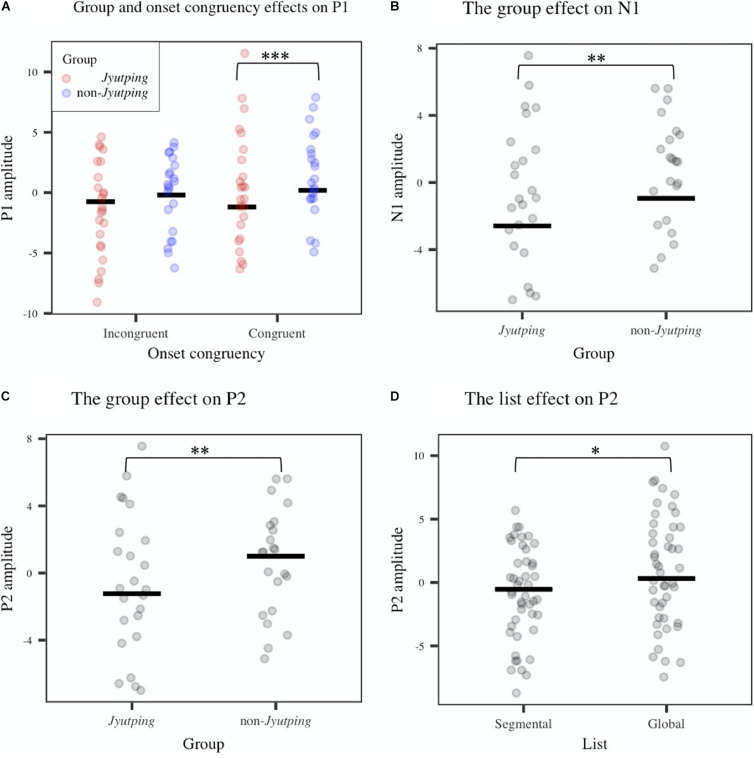
Averaged ERP amplitude in the P1 (0–93 ms), N1 (95–237 ms), and P2 (239–353 ms) time-windows obtained from the fronto-central electrode cluster (F3, Fz, F1, FC1, FC2, and Cz). **(A)** Group and onset congruency effects on P1. **(B)** The group effect on N1. **(C)** The group effect on P2. **(D)** The list effect on P2. The solid line denotes means estimated from the linear model while each dot represents individual ERP amplitude. Significance levels: *p < 0.05; **p < 0.01; ***p < 0.001.

**FIGURE 4:**
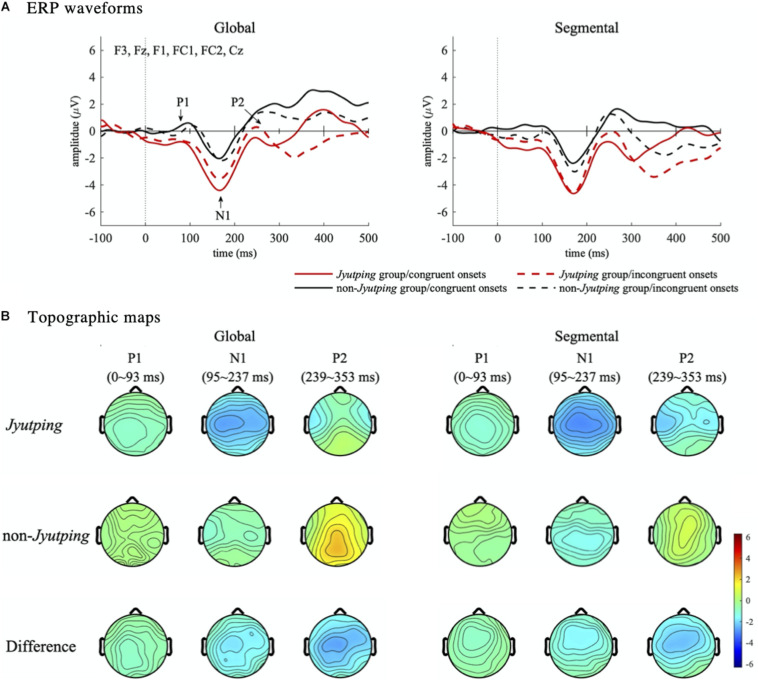
Event-related potential waveforms and topographic maps illustrating effects in the P1-N1-P2 time windows. **(A)** ERP waveforms. The ERP waveforms are averaged from the fronto-central electrode cluster (F3, Fz, F1, FC1, FC2, and Cz). The three auditory ERPs–P1, N1, and P2–are marked in the waveforms shown in the left panel. **(B)** Topographic maps. The topographies are pooled across congruent and incongruent onsets. Each map shows the mean amplitude in the three time-windows–P1 (0–93 ms), N1 (95–237 ms), and P2 (239–353 ms).

For auditory P1-N1-P2, which was analyzed based on the fronto-central electrode cluster, the ANOVAs revealed that the *Jyutping* group showed significantly more negative-going ERPs than the non-*Jyutping* group in P1 [*F*(1, 44) = 12.42, *p* < 0.01], N1 [*F*(1, 44) = 11.21, *p* < 0.01], and P2 time-windows [*F*(1, 44) = 8.42, *p* < 0.01]. There was also a significant interaction between *group* and *onset congruency* in the P1 time-window [*F*(1, 44) = 6.04, *p* < 0.05]. Post-hoc analysis with Holm-Bonferroni correction revealed that the *Jyutping* group exhibited more negative-going ERPs than the non-*Jyutping* group in the P1 time-window for congruent trials (*t* = –4.29, *p* < 0.001), but not for incongruent trials (*t* = –1.71, *p* = 0.09). Furthermore, the segmental list elicited more negative-going ERPs than the global list in the P2 time-window only [*F*(1, 44) = 5.85, *p* < 0.05]. The effects revealed by ANOVAs are displayed in Figure 3. The ERP waveforms averaged across all the selected electrodes in the fronto-central cluster and the topographic maps of the ERPs in the P1-N1-P2 time windows pooled across congruent and incongruent onsets are illustrated in Figure 4. Overall, the *group* effect seems to manifest as a sustained negativity that extends from the P1 to the P2 time-window.

**FIGURE 5:**
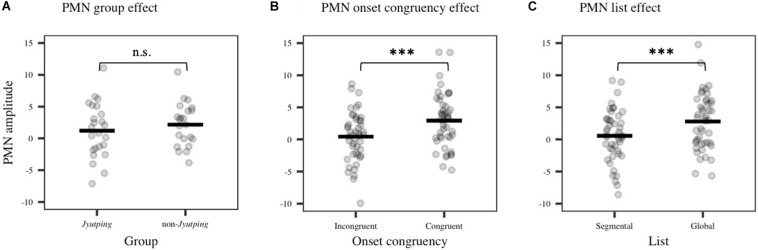
Averaged ERP amplitude in the PMN time-window (355–500 ms) obtained from the centro-parietal electrode cluster (P3, Pz, P4, CP1, CP2, and Cz). **(A)** PMN group effect (non-significant, n.s.). **(B)** PMN onset congruency effect. **(C)** PMN list effect. The solid line denotes means estimated from the linear model while each dot represents individual ERP amplitude. Significance levels: *p < 0.05; **p < 0.01; ***p < 0.001.

**FIGURE 6:**
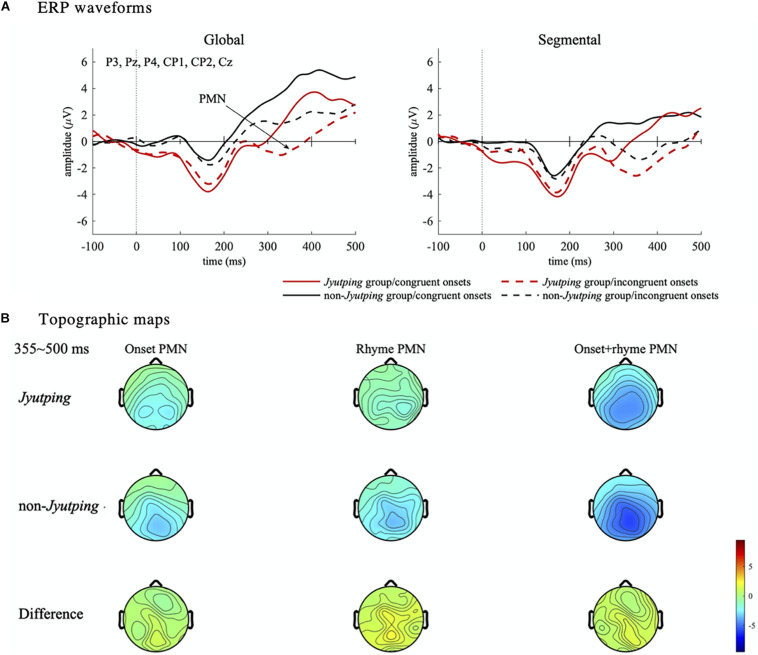
Event-related potential waveforms and topographic maps illustrating the effects in the PMN time window. **(A)** ERP waveforms. The ERP waveforms are averaged from the centro-parietal electrode cluster (P3, Pz, P4, CP1, CP2, and Cz). The PMN component is marked in the waveforms shown in the left panel. **(B)** Topographic maps. The topographies illustrate the mean amplitude of the isolated PMN component in the time-window from 355 to 500 ms. Three isolated PMNs are displayed to better delineate the PMN effect–onset mismatch (*incongruent onset/global list* minus *congruent onset/global list*), rhyme mismatch (*congruent onset/segmental list* minus *congruent onset/global list*) and rhyme+onset mismatch (*incongruent onset/segmental list* minus *congruent onset/global list*). Note the *congruent onset*/*global list* condition, which has completely identical paired pseudowords without any phonological mismatch, is the baseline to derive the PMN component.

For the PMN component which was analyzed based on the centro-parietal electrode cluster, there was a significant main effect of *list* [*F*(1, 44) = 15.15, *p* < 0.001], with larger negativity elicited in the segmental list than in the global list. We also found a significant effect of *onset congruency* [*F*(1, 44) = 28.31, *p* < 0.001], with larger negativity elicited by trials with incongruent onsets than with congruent onsets. No significant effect of *group* or its interactions with other factors were found (*ps* > 0.05). Thus, there is no evidence that the phonological mismatch detection process is modulated by the participants’ knowledge of *Jyutping*. The *onset congruency* effect reflected the mismatch between the onsets of the pseudowords in a pair. Furthermore, the *list* effect might reflect the detection of rhyme mismatch, even though the task focused on onset judgment. To further explore the PMN pattern, post-hoc pairwise comparisons with Holm-Bonferroni correction were performed among the four conditions collapsed across *Jyutping* and non-*Jyutping* groups, that is, the four combinations of onset and rhyme mismatch–*incongruent onset/segmental list* (different onsets and different rhymes), *congruent onset/segmental list* (same onset and different rhymes), *incongruent onset/global list* (different onsets and same rhyme) and *congruent onset/global list* (same onset and same rhyme). Compared with the *congruent onset/global list* condition, the other three conditions exhibited more negative ERPs in the PMN time-window (*p*s < 0.001). These results reaffirmed our prediction that PMN would be elicited for both onset and rhyme mismatches. Moreover, the *incongruent onset/segmental list* condition elicited larger negativity than the *congruent onset/segmental list* condition (*t* = –4.03, *p* < 0.001), and the *incongruent onset*/*global list* condition (*t* = –3.39, *p* < 0.01). This result suggested the largest PMN in the *incongruent onset/segmental list* condition with both onset and rhyme mismatches. However, the *congruent onset/segmental list* condition, and *incongruent onset/global list* condition did not differ from each other significantly (*t* = 0.25, *p* = 0.81), suggesting similar PMN amplitude for rhyme and onset mismatch. Thus, the additional analyses indicated cumulative effects of onset and rhyme mismatch on the PMN amplitude (least-square means in four conditions: *congruent onset/global list*: 4.04 μV; *incongruent onset/global list*: 2.33 μV; *congruent* onset*/segmental list*: 2.19 μV; *incongruent onset/segmental list*: 0.40 μV). The PMN effects revealed by ANOVAs are displayed in Figure 5. The ERP waveforms averaged across all the selected electrodes in the centro-parietal cluster are displayed in Figure 6A. The topographic maps of the three isolated PMNs induced by onset mismatch, rhyme mismatch and rhyme+onset mismatch are displayed in Figure 6B. As indicated by the topographic maps, the PMN has a centro-parietal distribution and, consistent with the statistical results, it has the largest amplitude in the rhyme+onset mismatch condition. However, no group differences can be observed.

## Discussion

We found that participants in the *Jyutping* group achieved higher accuracy than the non-*Jyutping* group in auditory onset phoneme judgment. This behavioral result is associated with the ERP pattern that the *Jyutping* group showed more negative-going ERPs in early auditory P1-N1-P2 than the non-*Jyutping* group. These findings provide further support for the claim that speech representations and processing at the phonemic level become more explicitly accessible after the acquisition of an alphabetic script (Morais et al., 1979; Ziegler and Goswami, 2005; Morais, 2021). The ERP findings provide further insight on the temporal dynamics of the previously reported enhanced phonetic/phonological processing in Chinese readers with alphabetic literacy (Read et al., 1986; de Gelder et al., 1993; Ho and Bryant, 1997; Cheung et al., 2001; Brennan et al., 2013).

The sustained stronger negativity observed in the *Jyutping* group that emerged from the P1 time-window (0–93 ms) and continued until the N1 (95–237 ms) and P2 time-window (239–353 ms) could be attributed to their enhanced ability to encode acoustic-phonetic information or to process phonological units at a more abstract fine-grained phonemic level. The P1-N1-P2 complex has been shown to encode phonetic features and index early stages of phonetic/phonological processing (Obleser et al., 2003; Tremblay et al., 2003; Toscano et al., 2010). For instance, the N1/N1m component has been reported to encode acoustic-phonetic features such as voice onset time (Toscano et al., 2010) and consonant/vowel place features (Obleser et al., 2003). Moreover, the neural generators of P1-N1-P2 lie in multiple auditory areas, like Heschl’s gyrus, PT and STG, that are relevant for auditory analysis of speech (Martin et al., 2008). These neural sources are broadly consistent with the brain regions recruited during phonetic/phonological processing (e.g., PT and STG), which show augmented activations to speech in competent readers of alphabetic scripts compared with preliterate children, illiterate adults or readers of logographic scripts (Dehaene et al., 2010, 2015; Brennan et al., 2013; Monzalvo and Dehaene-Lambertz, 2013). Thus, the current ERP findings support the view that the acquisition of an alphabetic script can lead to enhanced acoustic-phonetic processing at a lower auditory level or the functional reorganization of the higher-level phonological network that is responsible for encoding more fine-grained phonological units like phonemes (Castro-Caldas et al., 1998; Petersson et al., 2000; Dehaene et al., 2010, 2015; Brennan et al., 2013; Monzalvo and Dehaene-Lambertz, 2013).

An alternative or complementary account is that the negativity observed in the time-windows of early auditory evoked potentials for the *Jyutping* group also reflects enhanced attention/awareness processes. Note that the *Jyutping* group exhibited more negative ERPs in the P2 time-window, instead of larger (or more positive) P2 as predicted. Enlarged P2 has been frequently reported to be associated with enhanced auditory or phonological processing, for example, in musicians or after laboratory training on speech or musical sounds (Tremblay et al., 2001; Tremblay and Kraus, 2002; Shahin, 2011). One possible hypothesis is that the amplitude reduction in the P2 time-window for the *Jyutping* group may be related to a superimposed processing negativity (PN) wave, also known as negativity difference (Nd), or contingent negative variation (CNV), that reflects top-down attentional processes (Tecce, 1972; Näätänen et al., 1978; Näätänen, 1982; Näätänen and Picton, 1987; Lange et al., 2003; Baumann et al., 2008). These relatively low-frequency waves can overlap early auditory ERPs. For example, Baumann et al. (2008) found a similar low-frequency PN-like or Nd-like component for musicians, which was superimposed on auditory P1-N1-P2 in their pitch, sine wave and instrumental tone discrimination tasks. As argued by the authors, the enlarged N1-P2 *per se* in musicians probably reflects an increase of neural resources for auditory feature encoding. Yet, this enhancement may be distinct from a superimposed PN-like negative wave which reflects the plasticity of attentional control. They reasoned that if musical training leads to both enhanced positivity in P2 and negativity in a PN-like component, the different polarities of these two components could possibly explain their null effect of musical expertise in the P2 time-window. Consistent with the PN explanation, the overall group difference in our ERP data seems to manifest as a fronto-centrally distributed, prolonged negativity, which begins approximately at the stimulus onset and continues into later sensory/perceptual processing stages as indexed by auditory evoked potentials. Therefore, the attentional account captures the amplitude reduction in the P2 time-window or more generally the globally sustained negativity for the *Jyutping* group across the P1-N1-P2 time windows. Furthermore, the attentional account holds some support from neuroimaging studies demonstrating the engagement of the attentional network in meta-phonological tasks like auditory phoneme judgment (Burton et al., 2000). Literacy and phonological awareness skills have also been reported to modulate functional activations of the attentional network in a pseudoword repetition task which requires some meta-phonological skills (Castro-Caldas et al., 1998; Petersson et al., 2000). These tasks that tap meta-phonological skills might require participants’ abilities to shift their attention to phonology in general or more specifically to fine-grained phonological units like phonemes.

Besides the *group* effect, the behavioral data showed that the segmental list elicited more errors and slower responses than the global list, but these effects were only present for the trials with congruent onsets. These results suggested higher task requirements or greater difficulties for the segmental list (Burton et al., 2000). However, the segmental list is not uniformly more difficult than the global list. The trials in the *congruent onset/segmental list* condition are the most difficult probably due to the necessity of ignoring or inhibiting the relatively salient rhyme “difference” and focusing on the target onset dimension to arrive at a correct “same” onset response.

For ERP effects regarding *list* and *onset congruency*, the segmental list elicited more negative ERPs than the global list in the P2 time-window, probably reflecting higher demand on phonetic/phonological processing or attentional modulation in the segmental list. Another possibility is that the *list* effect in the P2 time-window was due to the partial overlap with the ensuing PMN component that is sensitive to the rhyme mismatch. The PMN component (355–500 ms) was modulated by both list and onset congruency differences, which can be explained by the rhyme and onset mismatch in the speech stimuli, respectively. A widely accepted theory is that the PMN represents phonological analysis of the acoustic features of speech sounds, which is affected by phonemic expectations established by prior contexts (Connolly and Phillips, 1994; Newman et al., 2003; Lafontaine et al., 2012; Zhang, 2018). Our finding that both onset and rhyme mismatches modulate the PMN amplitude is in line with this theory. Additionally, the PMN seems to index overall phonological mismatch in the speech signals regardless of its task relevance, i.e., the target onset dimension and the task-irrelevant rhyme dimension. Consistent with this idea, the PMN reported here is the strongest in the *incongruent onset/segmental list* condition, i.e., both onset and rhyme mismatches, although the behavioral results suggest that the *congruent onset/segmental list* condition should be the most difficult condition as discussed above.

Then, what could possibly explain the absence of a group difference for the PMN component? One explanation is that the locus of the influence of alphabetic literacy skills on phonemic processing is at an earlier acoustic-phonetic encoding stage as indexed by the P1-N1-P2, but not at a later phonological processing stage as indexed by the PMN. Another possibility is that the PMN *per se* is probably not specific to more abstract “phonemic” operations, like translating acoustics into phonemic categories, or detecting mismatch between phonemic expectations and incoming speech signals, as postulated by the current PMN theory (Connolly and Phillips, 1994; Newman et al., 2003; Lafontaine et al., 2012; Zhang, 2018). Instead, a mismatch between auditory traces in the working memory and the incoming speech signal might be sufficient to generate the PMN, regardless of the grain size of phonological representations.

We also predicted that there might be interactions among *group*, *list* and *onset congruency* in both the behavioral and ERP responses. Only the interaction between *group* and *onset congruency* in the P1 time window was consistent with this prediction. One possible explanation for this interaction effect is that congruent trials, especially those in the *congruent onset/segmental list* condition, might be more difficult and thus increase the attentional load or phonetic/phonological encoding demand at an early stage. However, further studies are needed to confirm this initial observation on the interaction between task difficulties or processing demands and the impact of alphabetic literacy on phonemic processing.

One potential limitation of the present cross-sectional study is that participants’ *Jyutping* skills might be correlated with or affected by other pre-existing skills, like their knowledge of the English alphabet. For example, the two groups of participants were matched on their general knowledge of English that was assessed by standardized English tests. Nevertheless, we could not completely rule out the possibility that English phonological awareness and alphabetic skills might be correlated with their Cantonese phonological awareness and *Jyutping* knowledge, and further contribute to the behavioral and neural differences observed between the two groups. Indeed, many aspects of the cross-language transfer effects of phonological awareness and literacy skills remain controversial (including the direction of such transfer effects: L1-to-L2 or L2-to-L1), and the discrepancies could be due to multiple factors (e.g., the phonological and orthographic similarity between two languages; Bialystok et al., 2005; Dodd et al., 2008; Deng et al., 2019). The current finding confirmed the behavioral advantage of alphabetic literacy skills in speech processing in one’s native language and provided further evidence on the underlying neuro-cognitive processes of such effects. Nevertheless, future research can employ a longitudinal design with an alphabetic literacy training paradigm and test participants’ alphabetic/phonological skills in different languages before and after training. This design would help to segregate the influences of L1 and L2 literacy knowledge and phonological awareness skills on L1 speech processing and explore their possible interactions during learning. Furthermore, with this design researchers can further demonstrate the causal link between alphabetic literacy skills and phonological awareness enhancement, and to assess both their language-specific and language-independent components.

## Conclusion

The major goal of the present study is to examine whether and how Chinese speakers’ phonological awareness and the temporal unfolding of its underlying neuro-cognitive processes are sensitive to alphabetic literacy. We showed that logographic Chinese readers with *Jyutping* expertise performed auditory onset phoneme judgment more accurately. For ERP responses, the *Jyutping* group exhibited more negative-going ERPs in early auditory P1-N1-P2 time-windows than the non-*Jyutping* group. The PMN component exhibited sensitivity to both onset and rhyme mismatches in the speech signals, but it was not modulated by alphabetic literacy skills. The early enhancement in the processing stream probably reflects the increased efficiency of phonetic/phonological encoding and the associated attention/awareness modulation.

## Data Availability Statement

The datasets presented in this study can be found in online repositories: https://osf.io/2pm7y/.

## Ethics Statement

The studies involving human participants were reviewed and approved by The Human Subjects Ethics Sub-committee of the Hong Kong Polytechnic University. The participants provided their written informed consent to participate in this study.

## Author Contributions

YZ: formal analysis, visualization, and writing–original draft. CP: conceptualization, methodology, and writing–review and editing. MB and DL: conceptualization. K-YL and L-YY: methodology and investigation. CZ: conceptualization, writing–review and editing, supervision, and funding acquisition. All authors contributed to the article and approved the submitted version.

## Conflict of Interest

The authors declare that the research was conducted in the absence of any commercial or financial relationships that could be construed as a potential conflict of interest.

## Publisher’s Note

All claims expressed in this article are solely those of the authors and do not necessarily represent those of their affiliated organizations, or those of the publisher, the editors and the reviewers. Any product that may be evaluated in this article, or claim that may be made by its manufacturer, is not guaranteed or endorsed by the publisher.
